# Vascular Illness, Cognition, and Subjective Aging: Examining the Vascular Hypothesis of Aging

**DOI:** 10.1093/geroni/igab046.2638

**Published:** 2021-12-17

**Authors:** Victoria Dunsmore, Shevaun Neupert

**Affiliations:** North Carolina State University, Raleigh, North Carolina, United States

## Abstract

Cognition relates longitudinally and cross-sectionally to physical and psychological health among older adults. The Vascular Hypothesis of Aging (Drewelies & Gerstorf, 2020) suggests that illnesses of a vascular nature (e.g., stroke, hypertension, severe varicose veins) negatively affect cognitive abilities. Awareness of age-related change (AARC) is also related to cognition. What is not known is whether the presence of a vascular illness and daily cognitive abilities interact to predict daily awareness of age-related changes. The purpose of this study is to examine the daily fluctuations of cognition, (i.e., memory failures) and their interaction with vascular illness to predict daily awareness of age-related changes. Data were analyzed from 104 participants (M age = 64.67, 60-90 years) who completed online self-report questionnaires. On Day 1, participants answered baseline questionnaires regarding presence of vascular illness, and on Days 2-9 completed measures regarding AARC losses and memory failures. Multilevel models revealed main effects of daily memory failures on awareness of age-related losses, such that on days with more memory failures, older adults reported more age-related losses. We also found a main effect for vascular illness, such that those with a vascular illness reported higher levels of daily age-related losses. We did not find a significant interaction between vascular illness and daily memory failures on daily reported age-related losses. Our results provide preliminary evidence that the vascular hypothesis of aging may also extend to perceptions of age-related changes. Future research could consider examining daily symptoms of vascular illness as they unfold over time.

